# Transmural collaborative care model for cardiovascular risk management and medication review in patients using antipsychotics in primary care (TACTIC): A study protocol of an incomplete stepped wedge cluster randomized trial

**DOI:** 10.1016/j.conctc.2024.101418

**Published:** 2025-01-09

**Authors:** Kirsti M. Jakobs, Karlijn J. van den Brule-Barnhoorn, Jan van Lieshout, Joost G.E. Janzing, Wiepke Cahn, Wietske Kievit, Steven Teerenstra, Maria van den Muijsenbergh, Marion C.J. Biermans, Erik W.M.A. Bischoff

**Affiliations:** a Radboud University Medical Center, Primary and Community Care Department Nijmegen, the Netherlands; b Zorggroep Onze Huisartsen B.V., Arnhem, the Netherlands; c Radboud University Medical Center, IQ Health Science Department, Nijmegen, the Netherlands; d Radboud University Medical Center, Psychiatry Department, Nijmegen, the Netherlands; e University Medical Center Utrecht, Psychiatry Department, Utrecht, the Netherlands; f Altrecht Science, Altrecht Mental Health Institute, Utrecht, the Netherlands; g Radboud University Medical Center, IQ Health Science Department, Section Biostatistics, Nijmegen, the Netherlands

**Keywords:** Primary health care, Antipsychotics, Cardiovascular risk, Collaborative care, Deprescribing, Off label

## Abstract

**Background:**

It is well established that patients with severe mental illness and those treated with atypical antipsychotics (AAPs) are at an increased risk of cardiovascular disease. However, primary care currently lacks adequate monitoring of AAP usage, its effects, and the associated cardiovascular risk. We have developed TACTIC, a transmural collaborative care model for patients using AAPs prescribed by the general practitioner (GP) to address the issues of potential overtreatment with AAPs and undertreatment for cardiovascular risk. TACTIC comprises three steps: an informative video for patients, a multidisciplinary meeting, and a shared decision-making consultation with the GP.

**Objectives:**

To evaluate TACTIC's effectiveness on cardiovascular risk and mental health and its cost-effectiveness.

**Methods:**

We will conduct an incomplete stepped wedge cluster randomized trial in the Netherlands.

40 GP-nurse clusters are randomized into four waves. Each cluster recruits adult patients (25–85 years), without prior diagnoses of dementia, delirium, or cardiovascular disease, for whom the GP prescribes AAPs. Every five months, a new wave starts with TACTIC. Measurements are taken before the intervention starts and every 5 months until the study concludes. Primary outcomes are cardiovascular risk and mental health as measured with the QRISK3 score and MHI5, respectively. The economic evaluation consists of two cost-utility analyses, one on the data collected alongside the trial and one based on a model extrapolating the trial data to a 10-year horizon. We will also evaluate the process of delivering TACTIC.

**Conclusion:**

This study will assess TACTIC's (cost)effectiveness and provide insights for successful delivery in general practice.

**Clinical trials registration:**

clinicaltrials.gov NCT05647980.

## Highlights

• Patients on antipsychotics are often discharged to primary care without ongoing monitoring of the benefits and risks.
• General practitioners are unlikely to change prescriptions of antipsychotics they did not initiate.
• TACTIC is an intervention aiming to address overtreatment with antipsychotics and undertreatment for cardiovascular risk.
• We will evaluate the (cost)effectiveness of TACTIC in an incomplete stepped wedge randomized controlled trial.

## 1 Introduction

Prescriptions of Antipsychotics (APs) are on the rise worldwide [1]. In the Netherlands, the number of users increased by 48 % from 2003 to 2022 [2]. APs are prescribed for psychiatric disorders, including schizophrenia, bipolar disorder, schizoaffective disorder, and major depressive disorder. These conditions are commonly referred to as severe mental illness (SMI). However, it is important to note that a considerable number of patients who are prescribed APs do not have an SMI diagnosis [3]. Jakobs et al. found that among patients in general practices who use antipsychotic medication, up to 68 % did not have a registered diagnosis of an SMI [4]. These patients are likely using APs off-label. Reasons for off-label prescription can be anxiety, agitation in dementia, sleep disorder, or challenging behavior of patients with an intellectual disability [5,6].

The use of APs, particularly atypical antipsychotics (AAPs), is associated with potentially serious adverse effects, including fatal arrhythmias and metabolic disturbances [7,8]. These adverse metabolic effects can develop in time and some APs are more likely to cause metabolic changes than others [7,9]. It is a well-known fact that individuals who suffer from severe mental illness tend to have a life expectancy that is 10–20 years shorter than the average person [10]. This is primarily due to an increased risk of mortality from cardiovascular disease (CVD) [8,11], which is caused by several factors. Patients with SMI have a higher incidence of lifestyle risk factors, such as poor diet, lack of exercise, stress, and smoking, which can contribute to the development of CVD [12,13]. Moreover, the adverse effects of AAPs are an independent cardiovascular risk (CVR) factor [7,14,15].

In the Netherlands, many patients on AAPs are discharged to primary care without a care plan for monitoring treatment effects, adherence, side effects, and management of CVR [4]. The increased CVR in patients taking AAPs can be managed in general practices, with lifestyle counseling as well as pharmacological interventions, in the same way as managing CVR in other patient groups [16].

However, GPs are not as familiar with adjusting AAPs use as psychiatrists [17], GPs and psychiatrists often do not collaborate in reducing CVR in patients using AAPs [17], and patients with mental illness participate less often in preventive care programs [18]. The latter is explained by psychiatric symptoms, fear, distrust in the health care system, and low priority [19]. Furthermore, patients with mental illness are less likely to receive standard care because stigma toward these patients influences physicians' health decisions [20]. This may affect the willingness to proactively invite patients with mental disorders using AAPs to monitor their CVR. As a result, when patients are referred to primary care in a stable phase, CVRM is lacking, and patients continue their AAP medication even if their risk has worsened over time.

**Fig. 1:**
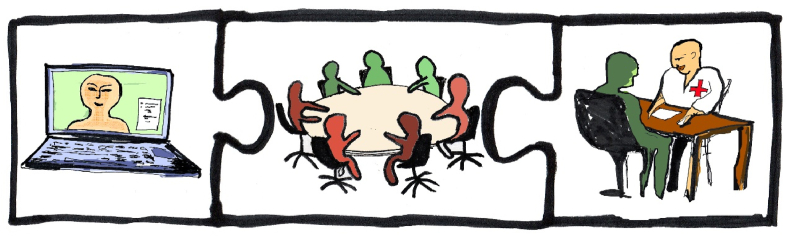
The TACTIC intervention consists of an informative video for patients, a multidisciplinary meeting, and a shared decision visit.

To improve AAP monitoring and to reduce cardiovascular risk in patients using AAP in general practice, we have recently developed an intervention named” **T**ransmural collaborative care model for the review of **A**ntipsy**C**ho**TIC**s” (TACTIC, see further Fig. 1 and the methods section).

To our knowledge, TACTIC is the first intervention focussing on medication review and primary prevention of cardiovascular disease in patients on AAPs in general practice. The TACTIC intervention aims to enhance healthcare services for a neglected population in Dutch primary care.

## 2 Study aims

The study aims to evaluate the impact of TACTIC on participants' health, assess the cost-utility of delivering TACTIC, and examine the process of delivery of TACTIC in the participating general practices.

## 3 Methods

### 3.1 Setting

This study will be conducted in general practices in the Netherlands.

### 3.2 Design

**Fig. 2:**
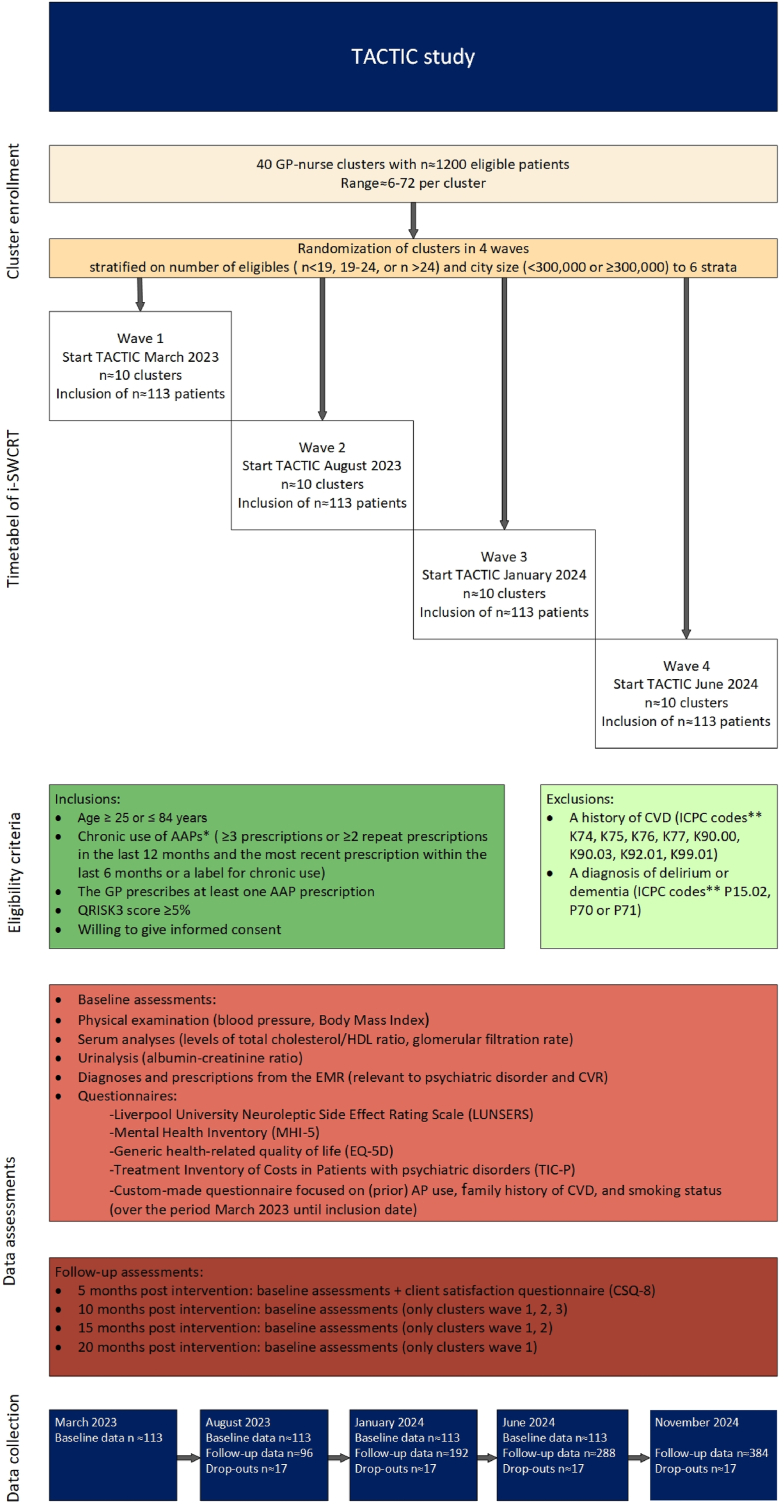
TACTIC study diagram. ∗The prescriptions from the ATC codes [22]. The ATC codes are similar to those in the QRISK3 algorithm as far as they are registered in the Netherlands: N05AX12, N05AD06, N05AH02, N05AE05, N05AH03, N05AX13, N05AH04, N05AX08, N05AE03, N05AX15, N05AX16 ∗∗The diagnoses from the ICPC codes [23]. Abbreviations: AAP, atypical antipsychotic; AP, antipsychotic; ATC, anatomical therapeutic chemical; CVD, cardiovascular disease; CVRM, cardiovascular risk management; CVD, cardiovascular disorder; EMR, electronic medical records; GP, general practitioner; HDL, high-density lipoprotein; ICPC, International Classification of Primary Care; i-SWCRT, incomplete stepped wedge cluster randomized trial; QRISK3, a tool to calculate the estimated CVD risk within the next 10 years for people aged between 25 and 84 without CVD.

For the evaluation of TACTIC, we have chosen an incomplete stepped wedge cluster randomized trial (i-SWCRT), implemented from March 2023 until November 2024 (see Fig. 2). The reason for not choosing a standard stepped wedge design, is the ensuing ethical problem of patients with an established high CVR who are withheld from treatment due to their GP's randomization in a standard stepped wedge design. For instance, if a patient is part of a cluster that belongs to wave four, according to a complete format, their first CVR screening will take place in March 2023 and will be repeated every five months until the intervention begins in June 2024. With an i-SWCRT, delivery of TACTIC, and initiation of CVR lowering strategies, would follow without delay in 6–8 weeks after the first CVR screening. Until the start of their wave, patients will continue to receive care as usual. Simulations to determine power showed that an i-SWCRT will provide sufficient power. This finding aligns with the general observation that i-SWCRTs offer nearly comparable power to complete SWCRTs [21].

We followed the SPIRIT guidance for reporting the content of this study protocol [24].

### 3.3 Eligibility

The criteria for patient inclusion and exclusion in the study are presented in Fig. 2. The criteria are based on their ability to calculate the patient's QRISK3 score [15], which is used to assess their CVR (see further section 3.8.1 Primary outcomes). Patients with a history of cardiovascular disease, including acute myocardial infarction, acute coronary syndrome, heart failure, ischemic stroke, transient ischemic attack, peripheral artery disease, aortic aneurysm, or any revascularization procedure such as percutaneous coronary intervention or coronary artery bypass grafting are excluded due to the inability to calculate their QRISK3 score. We made a deliberate choice to exclude patients with a low QRISK3 score for the TACTIC intervention and set the cut-off value at 5 % based on the findings of our pilot study [25]. We considered that for the multidisciplinary meetings to be useful, the risk had to be high enough to justify their effort, as perceived by participants and care providers. This means that a higher risk level would lead to a more meaningful discussion. However, we also wanted to avoid excluding all young people. Our pilot study [25] found that setting the limit at 10 % would result in such exclusion due to the strong correlation between the risk estimate and age. In the regular Dutch CVRM program, the CVR risk assessment is conducted using the SCORE calculation [16]. A QRISK3 score of 5 % is not comparable to a SCORE of 5 %, because the first indicates morbidity and mortality due to CVD and the latter only mortality. We chose QRISK3 above SCORE, because SCORE does not consider the additional risk associated with having an SMI or using an AAP and is not validated to assess the risk for patients with diabetes. For patients with diabetes, the dilemma regarding the use of AAP is even more urgent than for those without. For this study, we will use the QRISK3 score(10) to calculate the change in CVR specifically associated with SMI and AAP. We will invite patients with a QRISK3 ≥5 % to participate in the i-SWCRT. If the QRISK3 score is below 5 %, the patient does not meet the study criteria and will be cared for by their GP depending on regional agreements concerning CVRM.

### 3.4 Recruitment

#### 3.4.1 Recruitment of general practices

We will recruit GPs through a video shared through social media platforms and with primary care co-operatives. Dutch GPs are well organized in regional primary care co-operatives, which aim to provide high-quality chronic disease management in primary care for patients with diabetes mellitus, cardiovascular diseases, high CVR, COPD, asthma, mental health needs, and frailty in old age [[26], [27], [28]].

#### 3.4.2 Patient recruitment

An algorithm has been developed to identify eligible patients for this study [29]. The algorithm uses routine health data recorded by GPs in the electronic medical records (EMR). Participating GPs will use this algorithm on their EMR to generate a list of potentially eligible patients. The algorithm will consider patients who meet the study criteria and have an estimated QRISK3 score of at least 4 %, based on the available data in their medical records. As the algorithm could underestimate the CVR due to missing data, we chose 4 % as a more conservative cut-off than the 5 % that we use as the inclusion criterion. However, definite eligibility will be determined by a CVR screening in the practice. After the start of the cluster, all identified patients receive an invitational letter from their GP, informing them about the upcoming screening. Patients interested in participating are invited to contact the general practice. For patients who do not respond to the letter, the practice will call them by phone to invite them to assess their CVR. For the CVR assessment, all variables of QRISK3 are mapped using data from the EMR, supplemented with blood and urine tests, and questionnaires (See Table A2 in the Appendix for more information). The maximum number of patients per cluster is limited to 15, as the pilot study found this to be the maximum the cluster could effectively handle within the given timeframe.

#### 3.4.3 Patient consent

Once the patient is deemed eligible, the investigator will contact the patient to confirm their willingness to participate in the study. We also provide a patient information letter, with an “easy reading” version for patients with low literacy. The patient, along with their guardian if applicable, will be asked to provide written informed consent. If desired, the patient can contact an independent physician knowledgeable about the study but not involved in its execution.

### 3.5 Randomization

A cluster refers to a (group of) GP(s) who work(s) with one nurse. If a large practice has two nurses for its CVRM program, then the practice will have two clusters. The GPs who collaborate with the same nurse are added to that nurse's cluster. We have decided to randomly assign the GP-nurse clusters to avoid bias due to practice characteristics and contamination caused by GPs and nurses becoming aware of the consequences of using AAP. This awareness can influence how they treat patients who are using AAP, which could in turn affect the baseline measurements. Contamination in a practice consisting of two clusters is limited because such cases are exceptional. The clusters will be randomized to either of the four waves, as shown in Fig. 2. The randomization process is stratified based on two factors. The first factor is the number of eligible patients on the list of each GP in the cluster (<19, 19–24, >24). The second factor is the population size of the city where the practice is located (<300,000 or ≥300,000). The stratification of the city size will reduce the potentially diluting effect of metropolitan problems or regional interventions concerning CVRM. A minimization program is used to stratify with a random element and ensure balance in the allocation of the two factors over the waves. Blinding of the cluster as to their moment of implementing TACTIC was not possible. Due to the cross-over character, each participant will receive the intervention at some point in the study.

### 3.6 Intervention

TACTIC is a one-time intervention that takes place in the general practice setting (see Fig. 1). The design resulted from a project among healthcare professionals and patients in the region of Arnhem [30]. We have recently conducted a pilot study and found that the delivery of TACTIC in general practice was feasible [25].

TACTIC entails three consecutive steps in addition to usual care.

Step 1: All participating patients are shown an information video to inform them about the upcoming multidisciplinary meeting. The information video aims to motivate and prepare patients (and their carers) to participate in the multidisciplinary meeting. All healthcare professionals who take part in the multidisciplinary meeting will introduce themselves. The video will last 10–15 min and will be tailored for various multidisciplinary meeting settings.

Patients are encouraged to meet with their pharmacist or a person with a lived experience of an SMI to prepare for the multidisciplinary meeting, as was advised by participants in our pilot study.

**Fig. A1:**
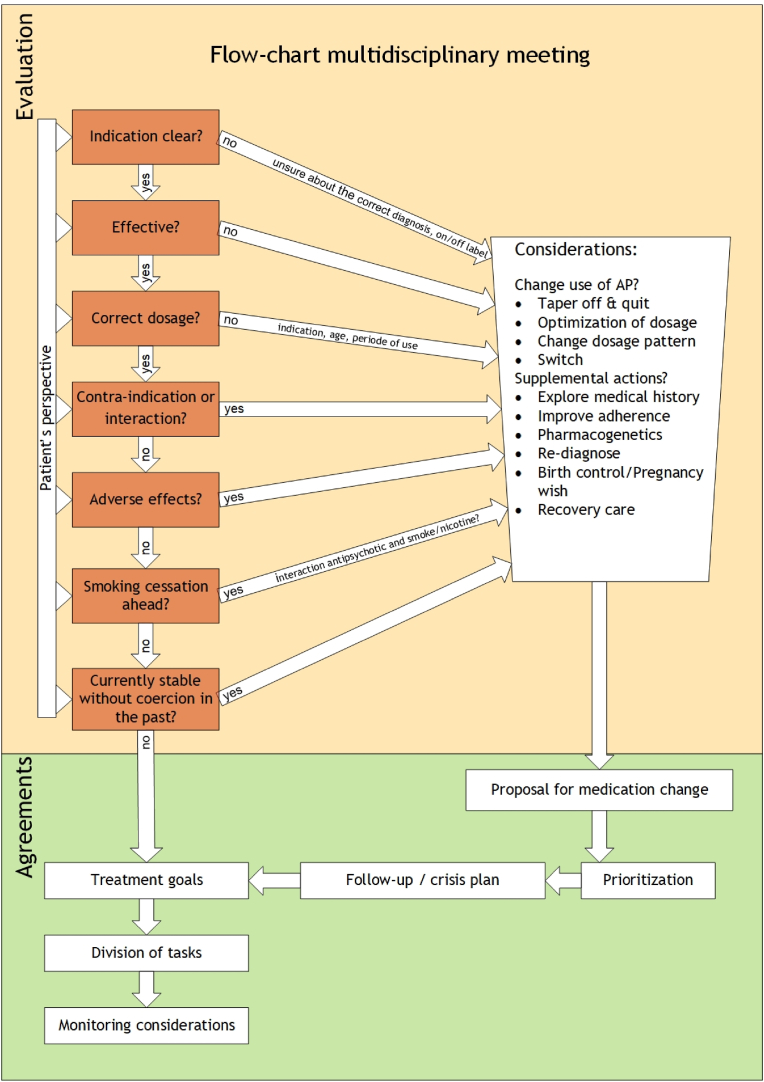
The structure of the multidisciplinary meeting.

Step 2: For every patient, a multidisciplinary meeting of 15 min will be conducted. The GP will send the relevant medical information to the psychiatrist at least one week before the meeting to allow for preparation. Most GPs open a so-called ‘digital consultation’ for the psychiatrist, which provides access to specific parts of the EMR of the GP. This includes the baseline assessments (see Fig. 2), the results of any advice provided by the pharmacist (if applicable), and the most recent correspondence of secondary mental health care (if applicable). During the multidisciplinary meeting, the participating patient (and carer), the general practitioner, a psychiatrist, the primary care nurse, and a person with a lived experience of an SMI will discuss the patient's AAP use following a structure of topics, shown in Appendix Figure A1. Additionally, all elevated CVR factors will be addressed. The multidisciplinary meeting will provide a set of personalized treatment recommendations, including advice on AAP use (e.g., continuation, deprescribing, or switching) and reducing other CVR factors (e.g., lifestyle changes, hypertension treatment, cholesterol regulation).
Step 3: Following the multidisciplinary meeting, the patients will have a scheduled appointment with their GP within a week. During this visit, they will work together to devise a customized action plan based on the recommended treatment options. The plan will outline the tasks and responsibilities of different healthcare providers. Potential actions are altering AAP use, initiating antihypertensive medication or statins, referral to the chronic care nurse for example for smoking cessation, referral to the primary care mental health nurse, the dietician, the physical therapist, or a lifestyle coach. After Step 3, the nurse will typically assess the plan's effectiveness and monitor it in the future. If necessary, other healthcare providers may be consulted or involved

### 3.7 Participant compensation

All costs patients will incur for the laboratory measurements will be reimbursed.

### 3.8 Outcome measures

#### 3.8.1 Primary outcomes

We defined two primary outcomes, namely the change in CVR and the change in mental health status. For the CVR assessment, we use the QRISK3 score [15]. The QRISK3 is the preferred algorithm for assessing CVR according to the NICE guidelines for Cardiovascular Disease [31]. The algorithm requires information such as ethnicity, the use of AAP, and relevant diagnoses such as SMI to calculate CVR (see Table A2 for QRISK3 variables). SMI includes schizophrenia, bipolar disorder, psychosis, and moderate/severe depression. However, in the Netherlands, the degree of severity cannot be deduced from the EMR. The CVR of patients with mild depression would be overestimated and therefore depression will not be classified as an SMI in our analysis.

QRISK3 is developed as a screening instrument. We will make the following adjustments to make the QRISK3 score algorithm useable to measure change. For age, we will use age at baseline (T0) at all measurement points. In the QRISK3 algorithm blood pressure treatment is considered a risk factor for CVR, as the patient on blood pressure treatment is considered a known case of hypertension. As a result, during the trial, the QRISK3 score may increase if the first prescription for blood pressure treatment is due to the intervention. To measure a change in QRISK3-score as correctly as possible, we will disregard the variable ‘blood pressure treatment’ in the algorithm during follow-up. If a participant reports a family history of cardiovascular disease (CVD) at any point during the trial, we will consider this information at all measurement points. This is because a positive family history of CVD is a characteristic that is not dependent on time and influences the patient's cardiovascular risk (CVR) before the status of the family history is known.

Unfortunately, the Townsend deprivation score cannot be applied to the Dutch population. Therefore, it will be set to zero as instructed on the website https://qrisk.org [32], indicating neither deprivation nor affluence. The QRISK3 score can range from 0 to 100 %. We consider a decrease of 2.5 % points as clinically relevant (number needed to treat = 40).

For the mental health status, we use the five-item version of the Mental Health Inventory (MHI-5) [33]. The MHI-5 ranges from 0 to 100, where a score of 100 equals perfect mental health. In the absence of an established minimum clinically important difference (MCID), we followed Cohen's interpretation of a small effect defined as 0.2 x the SD. In our pilot study, 17 patients with a QRISK3 ≥5 % had an SD of 15.2 for the MHI-5. Therefore, we will consider an increase of 3 points for an individual as clinically important [34]. See further Table A2. in the Appendix.

#### 3.8.2 Secondary outcomes

Five secondary outcome measures will also be examined: change in the generic health-related quality of life, as measured with the EuroQol-5D-5L [35]; change in side effects of antipsychotic medication, as measured with the Liverpool University Neuroleptic Side Effect Rating Scale (LUNSERS) questionnaire [36]; participants' satisfaction with TACTIC, as measured with the 8-item Client Satisfaction Questionnaire (CSQ-8) [37]; change in QRISK3 score with the Dutch deprivation score entered in the algorithm; and change in risk score as a proportion of the maximum achievable change in QRISK3 score.

Additionally, we will be measuring the delivery and uptake of TACTIC, including the number and content of advice given and the follow-up actions taken. Moreover, we will be examining healthcare utilization and productivity losses (TIC-P) [38], while also exploring how much TACTIC was used by the participating practices and identifying the factors that contribute to its successful delivery.

To provide a clear description of our measurements and questionnaires, we have included Table A2, which outlines the scores, ranges, and relevance of each.

### 3.9 Planned statistical analysis

All measured data will be assembled in a computer database and analyzed using SPSS 29.

#### 3.9.1 Descriptive statistics

Descriptive analyses will be performed to describe the patient's characteristics at inclusion across the waves. Mean and standard deviation (SD) or median and interquartile range for continuous variables and numbers and percentages for categorical variables will be presented.

#### 3.9.2 Analyses for primary outcomes

The effect of treatment on the outcome measures measured at 5, 10, 15, and 20 months of follow-up will be analyzed with mixed three-level linear or logistic regression, considering that the times of measurement are clustered within patients, and patients within general practices. Random effects for cluster and patient nested within a cluster are used to capture the correlation of patients within clusters and the correlation of measurements within patients. To test the effect of the intervention we will use a model with intervention “off” in the first measurement and “on” in the following measurements of a patient. A value of p < 0.05 will indicate statistical significance for all analyses based on two-sided testing. The change in MHI-5 will only be tested if the first primary outcome, the change in QRISK3, is statistically significant.

#### 3.9.3 Sample size calculation for primary outcomes

The sample size calculation for a power of 80 % (see Appendix A3 for the formula) is based on the following findings and expectations. Our pilot study showed an SD of 12 for a single QRISK3 measurement, along with a mean reduction of 1.9 points in the QRISK3 score for patients who had a baseline QRISK3 score of ≥5 % (unpublished results of our pilot study). This was after a 3-month follow-up period with suboptimal intervention conditions, including inadequate preparation of participating patients, leading to unclear expectation management. We expect that in the trial, after the optimization of the TACTIC procedures, we will be able to detect a reduction of a clinically relevant mean QRISK3 score of at least 2.5 points in the intervention group, compared to the control condition. 500 simulated trials indicate that 4 waves, 32 clusters with 12 patients each, are needed to provide a power of at least 80 %, given an SD of 12, an intra-cluster correlation (ICC) of 0.10, a (test-retest) reliability on patient level of 0.95, assuming a reliability on cluster level of 1, and an α of 0.05. We chose a drop-out rate of 15 %. This is a considerate choice given the rates observed in our pilot study of 20 % (based on a selection of participants with a QRISK3≥5 %) and the drop-out rate of less than 10 % in a UK primary care study evaluating a comparable intervention in a similar patient group [39]. In our trial, we have changed our approach from the pilot study. Now, participants are encouraged to meet with their pharmacist or a person with lived experience to prepare for the multidisciplinary meeting. This change in approach is aimed at reducing the number of dropouts compared to those in the pilot study. The required total number of remaining participants will be 384 patients.

#### 3.9.4 Additional analyses

We will perform two additional analyses to support the primary analysis. In both additional analyses, we will use more data from the included patients in the current i-SWCRT.

1. We will use variables of QRISK3 found in the EMR of participating patients of waves 2, 3, and 4, which may be measured as routine care between the trial start date (01-March-2023) and the date of inclusion, and data on smoking status as collected in the questionary at baseline.
2. QRISK3 variables of the period between the trial start date and the date of inclusion that are missing in the EMR will be imputed.

Patients eligible at enrolment for their wave may be non-eligible at the start of the study. We will exclude these patients for these analyses.

#### 3.9.5 Economic evaluation

The cost-utility of TACTIC compared to usual care will be performed alongside the clinical trial and will comprise a medical and societal perspective. Effectiveness will be expressed as Quality-adjusted life year (QALY) estimated according to the trapezium rule with utilities derived from the EQ5D5L questionnaire [35]. At patient level, volumes of care related to treatment of underlying diseases of this patient group, costs related to performing TACTIC, medication costs, and productivity losses will be measured using data extraction from the electronic medical patient files or otherwise by the TIC-P Questionnaire [38]. For further explanation see Appendix A4.

### 3.10 Research ethics

This study will be conducted according to the principles of the Declaration of Helsinki of 2013. Ethical approval for this study was waived by the local Medical Research Ethics Committee Arnhem/Nijmegen (file number 2022–15835).

## 4 Discussion

There is a lack of information regarding the effectiveness of CVR-reducing interventions in primary care patients who are taking antipsychotics. The TACTIC intervention is one of the first collaborative care interventions to address the issues of overtreatment with AAPs and undertreatment for cardiovascular risk in primary care. TACTIC offers personalized advice and involves patients in their meetings. The i-SWCRT design enables CVR-lowering strategies without delay for all participants. It ensures a high level of evidence while requiring fewer participants than in a classic randomized controlled trial. This complex intervention and its study design were carefully considered based on the results of our pilot study [25].

It is important to note that our study will possibly have limitations that should be considered. Firstly, GPs and psychiatrists must be able to safely share relevant patient information using a digital system that is available locally. However, it may be difficult to implement this approach in regions that do not have access to such a system. Secondly, it is expected that enrolling an appropriate number of patients in the study may be difficult due to the characteristics of this hard-to-reach group [18,19]. Our approach to enrolling patients in the study involves requesting their GP to invite them for CVR screening and then inviting them to participate in the study after explaining the screening results. To make the enrolment process easier, we will provide an additional version of the patient information that is easy to read. Additionally, we will emphasize to patients that they have complete freedom to decide whether they want to follow the advice given and address any concerns about changes in medication. We reduced the risk of selection bias as much as possible by recruiting GPs in multiple regions in the Netherlands and by randomizing the list of eligible patients before inclusion. Yet, there may be a risk of selection bias among both.

In conclusion, this study will assess TACTIC's (cost)effectiveness and provide insights for successful delivery in general practice. Collaboration during a multidisciplinary meeting can enhance awareness and promote the exchange of knowledge among general practitioners, psychiatrists, nurses, and individuals with a history of severe mental illness. This can ultimately improve the quality of care provided to other patients.

## Data management

Since the data is pseudonymized, data will be published in the Radboud Data Repository (RDR) under restricted access. The license applied is by the Creative Commons Attribution Non-Commercial (CC BY-NC). This license allows for non-commercial reuse only with permission of the corresponding author and proper credit given.

## CRediT authorship contribution statement

**Kirsti M. Jakobs:** Writing – review & editing, Writing – original draft, Visualization, Project administration, Methodology, Funding acquisition, Conceptualization. **Karlijn J. van den Brule-Barnhoorn:** Writing – review & editing, Project administration, Methodology, Conceptualization. **Jan van Lieshout:** Writing – review & editing, Methodology, Funding acquisition, Conceptualization. **Joost G.E. Janzing:** Writing – review & editing, Methodology, Funding acquisition, Conceptualization. **Wiepke Cahn:** Writing – review & editing, Methodology, Funding acquisition, Conceptualization. **Wietske Kievit:** Writing – review & editing, Methodology, Funding acquisition, Conceptualization. **Steven Teerenstra:** Writing – review & editing, Methodology. **Maria van den Muijsenbergh:** Writing – review & editing, Supervision. **Marion C.J. Biermans:** Writing – review & editing, Methodology, Funding acquisition, Conceptualization. **Erik W.M.A. Bischoff:** Writing – review & editing, Supervision, Project administration, Methodology, Funding acquisition, Conceptualization.

## Funding

This work was supported by Care research and the Medical Sciences domain of the Dutch Research Council (10.13039/501100001826ZonMw) [project number 1010021190004].

## Declaration of competing interest

The authors declare that they have no known competing financial interests or personal relationships that could have appeared to influence the work reported in this paper.

## Figures and Tables

**Table A2:** TACTIC trial outcome measures and schedule of measurements

Topic	Construction	Description	Timeline∗
Mental health	Primary psychiatric diagnosis	Diagnosis in the EMR.	0
	Mental health questionnaire	The Mental Health Inventory, a five-item version (MHI-5), is a measure of mental health [33]. The MHI-5 is a derivative of the 36-item short-form health survey, the SF36, and assesses symptoms of depression and anxiety, loss of behavioral or emotional control, and psychological well-being in the prior four weeks. The MHI-5 ranges from 0 to 100, where a score of 100 equals perfect mental health. A score of 60 or higher is seen as good mental health, whereas a score below 60 is seen as poor mental health.	0, 5, 10, 15, 20
AP	AAP prescription	EMR concerning actual use, prescription in the past (until 5 years back), prescriber GP or psychiatrist, on- or off-label, change in daily dose, stop of AAP.	0, 5, 10, 15, 20
	Adverse effects AP questionnaire	The Liverpool University Neuroleptic Side Effect Rating Scale (LUNSERS) is a self-rating scale for measuring the side effects of AP medications [36]. LUNSERS was developed by researchers within the University of Liverpool to indicate the extent of side effects experienced by patients medicated with neuroleptic drugs. The scale consists of 41 known side effects of neuroleptics. Each ‘side-effect’ listed is scored on a five-point rating scale of 0–4, i.e., 0 = ‘Not at all’ and 4 = Very much. A total score can be calculated by adding all item scores and then be graded into ‘very low’, ‘low’, ‘average’, ‘high’, and ‘very high’, based on the percentiles. A clinically relevant improvement of at least the minimal clinically important difference (MCID), is defined as 0.5 x SD of the baseline mean.	0, 5, 10, 15, 20
	Participants' perception of AAP pros and cons questionnaire	A custom-made questionnaire on AAP use with 10 questions which consists of 2 open questions about the reason for the use and the effectivity of the use, and 8 yes/no questions concerning health workers involved (psychiatrist, coach), pregnancy, safety (drug addiction, restraining measures, suicide attempt, relapse prevention plan, presence of (informal) caregiver in case of change in medication).	0
QRISK3	EMR, demographic	Age, gender, and financial records that are indicative of socioeconomic status.	0, 5, 10, 15, 20
	EMR, medication	Blood pressure treatment, steroid tablets, treatment for erectile dysfunction, or AAPs.	0, 5, 10, 15, 20
	EMR, diagnostic	Levels of total cholesterol/HDL ratio, glomerular filtration rate, albumin-creatinine ratio, systolic blood pressure, variability of blood pressure (standard deviation), and Body Mass Index.	0, 5, 10, 15, 20
	EMR, diseases, and disorders	Having a diagnosis of severe mental illness (this includes schizophrenia, bipolar disorder, and moderate/severe depression), diabetes mellitus, chronic kidney disease, atrial fibrillation, migraines, rheumatoid arthritis, systemic lupus erythematosus, or erectile dysfunction.	0, 5, 10, 15, 20
	EMR, diagnostic, and a risk factor questionnaire	Additional 7 questions concerning ethnicity, smoking status, a history of premature coronary heart disease in a first-degree relative, education, and marital state.	0, 5, 10, 15, 20
	Proportion of patients with a QRISK3 change of ≥2.5 %	The proportion of patients who reach an absolute risk reduction of 2.5 % at the end of follow-up, which we consider as a clinically relevant change.	5, 10, 15, 20
	Proportional risk reduction of QRISK3	The proportional risk reduction is defined as ‘the change in QRISK3 score as a proportion of the maximum achievable change in QRISK3 score’. To give an example: a patient has a 10-year CVR of 20 %, but after perfectly optimizing all changeable risk factors, can reach a 10-year cardiovascular risk of 10 %. When, at the end of follow-up, the patient reaches a QRISK3 score of 17 %, this means the patient reached a 3 % absolute risk reduction (i.e., 20 %–17 %), and a 30 % proportional risk reduction (i.e., 3 %/(20 %–10 %)).	5, 10, 15, 20
	QRISK3 with a modified Townsend Deprivation Score	The Townsend Deprivation Score does not apply to the Dutch population. We want to perform a secondary analysis on the QRISK3 score in which we use the Dutch equivalent of deprivation. In the Netherlands, we use a deprivation index for patients with low socioeconomic status, based on employment, income, and percentage of non-western immigrants, “achterstandsindex" [40]. This deprivation index applies to approximately 10 % of the Dutch population. We decided to apply the Townsend Deprivation Score at p20 (below which are the 20 % most deprived of the British population) for the 10 % most deprived patients in our population. By choosing p20 instead of p10, we stay on the safe side and might underestimate the effect of deprivation, but we consider this more important than overestimating its impact.	
Quality of life	Quality of life questionnaire	Health-related quality of life will be measured with the EuroQol-5D-5L (EQ-5D) [35]. This instrument is available in a validated Dutch translation. The EQ-5D comprises five domains: mobility, self-care, usual activities, pain/discomfort, and anxiety/depression. The EQ-5D index is obtained by applying predetermined weights to the five domains and ranges from 0 to 1, where a score of 1 equals perfect health.	0, 5, 10, 15, 20
Patient Satisfaction	Satisfaction with care questionnaire	The 8-item Client Satisfaction Questionnaire (CSQ-8) to measure participants' satisfaction with care [37] has a 4-point Likert scale. The sum of 8 sub-scores about different aspects of received care can vary between 8 and 32. Higher scores mean higher satisfaction. This questionnaire is recommended for use in psychiatric patients and has a Dutch-validated version [41].	5
Delivery of TACTIC	EMR. Records concerning plans	Number and percentage of participants who watched the video (step 1 of TACTIC)	5
		Dates, numbers, percentage of attendance, and number and content of advice of multidisciplinary meetings (step 2 of TACTIC).	5
		Dates, percentage of attendance, and content of individualized treatment plans of shared decision visits (step 3 of TACTIC).	5
		Date of follow-up visit with the nurse, and type of advice followed up or executed.	5, 10, 15, 20
		Nurse mental health or nurse CVR case manager.	5
		Numbers and reasons for drop-outs.	0, 5, 10, 15, 20
		Numbers and types of (serious) adverse events.	0, 5, 10, 15, 20
Costs of intervention	Health utilization questionnaire	Healthcare utilization is measured with the Dutch version of the Treatment Inventory of Costs in Patients with Psychiatric Disorders (TIC-P) questionnaire [38].	0, 5, 10, 15, 20
	EMR, finance, and correspondence	Costs related to health care, such as medication, visits to the general practice, and visits to relevant medical specialists.	0, 5, 10, 15, 20

AAPs, atypical antipsychotics; APs, antipsychotics; EMR, electronic medical records; GP, general practitioner; HDL, High-Density Lipoprotein.

## Data Availability

Data will be made available on request.

## Appendix

The flow-chart resulted from a project among healthcare professionals and patients in the region of Arnhem [30]

## Appendix A3 The sample size calculation formula

The standard deviation of the difference in QRISK3 score was 3.4 and the standard deviation of a single measurement was twelve. This means there is a high (test-retest) reliability $r_{s}$ on the patient level, calculated via the relation:

$$rs=1-{\left(SDdiff/SD\right)}^{2}\;/\;2\approx 0.\;95$$

## Appendix A4 Economic evaluation

The standard cost prices from the ‘Dutch Guidelines for Cost Analyses’ and www.medicijnkosten.nl will be used. For units of care where no standard prices are available real cost prices will be determined based on full cost pricing. In the end, volumes of care will be multiplied by the cost prices for each volume of care to calculate costs.

Ultimately, the incremental costs will be related to the incremental QALYs expressed by the incremental cost-utility ratio (ICUR). Due to the stepped wedge design of the study, a multilevel model with time as a covariate will be used to analyze net monetary benefit (NMB) values to estimate the Incremental NMB (INMB). To estimate the uncertainty surrounding the ICUR, this regression model will be bootstrapped with one thousand replications. Results will be presented as means with 95 % percentiles, and graphically with a cost-effectiveness plane (raw data) and a willingness to pay (WTP) curve with varying WTP levels. Because the effects of better CVRM lie beyond what is possible to measure during the follow-up period of the trial, we will perform a second cost-effectiveness analysis in which costs and disutility related to cardiovascular events will be incorporated in a decision analytic modeling study. The QRISK3 score measured at the end of follow-up in this trial gives a patient a specific 10-year risk of CVD and will be multiplied by the cost and disutility related to specific cardiovascular events using a Markov Model [42]. Also, the management of CVR with for instance statin use will be included in the model. The costs and disutility related to CVD will be derived from the established CE model in the field of CVRM published in the literature. The influence of parameter uncertainty in this model will be explored by probabilistic sensitivity analysis with a Monte Carlo simulation (n = 1000).
